# Supplementary material for: Induction of Phlorotannins and Gene Expression in the Brown Macroalga Fucus vesiculosus in Response to the Herbivore Littorina littorea
Source: Mar Drugs. 2021 Mar 26;19(4):185. doi: 10.3390/md19040185 (PMC8067260; doi:10.3390/md19040185)
Supplement: Supplementary file 1 [file marinedrugs-19-00185-s001.pdf]

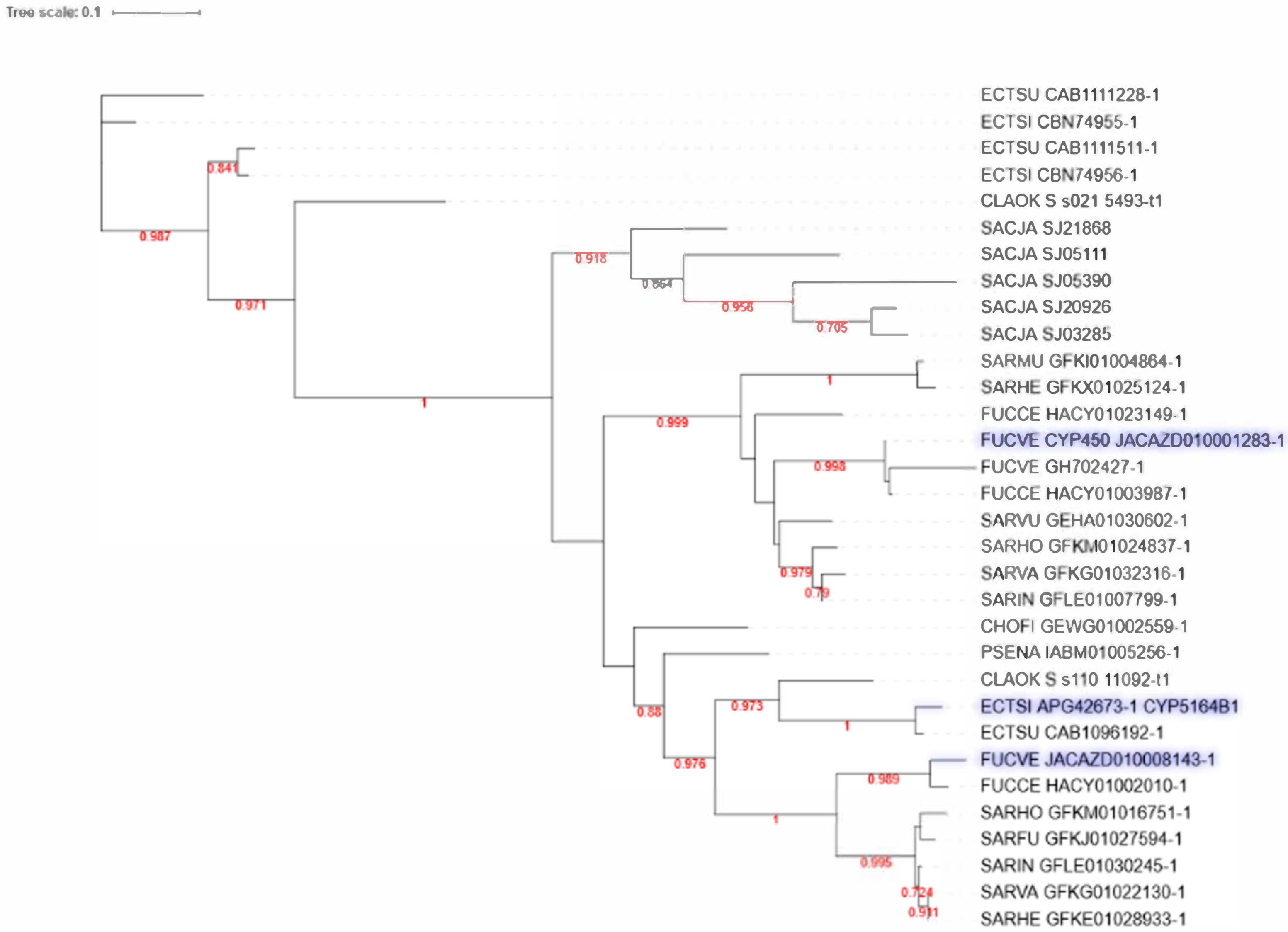

Figure S1. Dendrogram representation of the phylogenetic tree for homologous CYP450 sequences of 15 brown algal species.
The names of the sequences are composed of the 5 letters species code followed by the accession number of the blast hits. *Chorda filum* (CHOFI), *Cladosiphon okamuranus* (CLAOK)
Ectocarpus siliculosus (ECTSI), *Ectocarpus subulatus* (ECTSU), *Fucus ceranoides* (FUCCE), *Fucus vesiculus* (FUCVE), *Pseudochorda nagaii* (PSENA), *Saccharina japonica* (SACJA), *Sargassum fusiforme* (SARFU), *Sargassum hemiphyllum* and *Sargassum henslowianum* (SARHE), *Sargassum horneri* (SARHO), *Sargassum integerrimum* (SARIN), *Sargassum muticum* (SARMU), *Sargassum vachellianum* (SARVA), *Sargassum vulgare* (SARVU). Branch supports are indicated in red color. The biochemically characterized *E. siliculosus* epoxyalcohol synthase (CYP5164B1) and the *F. vesiculosus* representatives are highlighted in purple.
